# Fabrication and Characterization of Tantalum–Iron Composites for Photocatalytic Hydrogen Evolution

**DOI:** 10.3390/nano13172464

**Published:** 2023-08-31

**Authors:** Xiuru Yang, Anurag Roy, Mansour Alhabradi, Manal Alruwaili, Hong Chang, Asif Ali Tahir

**Affiliations:** 1 Solar Energy Research Group, Environment and Sustainability Institute, Faculty of Environment, Science and Economy, University of Exeter, Penryn Campus, Cornwall TR10 9FE, UK; xy328@exeter.ac.uk (X.Y.); ma943@exeter.ac.uk (M.A.); ma942@exeter.ac.uk (M.A.); 2 Faculty of Environment, Science and Economy, University of Exeter, Exeter EX4 4QF, UK; h.chang@exeter.ac.uk

**Keywords:** FeTaO_4_, photocatalytic hydrogen evolution, Ta_2_O_5_/FeTaO_4_, solar hydrogen, water splitting, photocatalyst

## Abstract

Photocatalytic hydrogen evolution represents a transformative avenue in addressing the challenges of fossil fuels, heralding a renewable and pristine alternative to conventional fossil fuel-driven energy paradigms. Yet, a formidable challenge is crafting a high-efficacy, stable photocatalyst that optimizes solar energy transduction and charge partitioning even under adversarial conditions. Within the scope of this investigation, tantalum–iron heterojunction composites characterized by intricate, discoidal nanostructured materials were meticulously synthesized using a solvothermal-augmented calcination protocol. The X-ray diffraction, coupled with Rietveld refinements delineated the nuanced alterations in phase constitution and structural intricacies engendered by disparate calcination thermal regimes. An exhaustive study encompassing nano-morphology, electronic band attributes, bandgap dynamics, and a rigorous appraisal of their photocatalytic prowess has been executed for the composite array. Intriguingly, the specimen denoted as 1000-1, a heterojunction composite of TaO_2_/Ta_2_O_5_/FeTaO_4_, manifested an exemplary photocatalytic hydrogen evolution capacity, registering at 51.24 µmol/g, which eclipses its counterpart, 1100-1 (Ta_2_O_5_/FeTaO_4_), by an impressive margin. Such revelations amplify the prospective utility of these tantalum iron matrices, endorsing their candidacy as potent agents for sustainable hydrogen production via photocatalysis.

## 1. Introduction

Due to the continuous accumulation of greenhouse gases in the atmosphere, caused mainly by human activities, extreme weather phenomena such as hurricanes, heatwaves, floods, and wildfires are occurring more frequently and with greater intensity [1]. The rise in this extreme weather has highlighted the significance of producing and using hydrogen fuel as a cleaner and more reliable energy source [2]. Governments, researchers, and businesses are stepping up their efforts to unlock the potential of hydrogen in our everyday lives and to incorporate it into our energy systems due to the urgency to mitigate climate change and advance energy sustainability [2,3]. Photocatalytic hydrogen evolution has emerged as a promising path in this quest, leveraging the extraordinary capabilities of particular materials known as photocatalysts to harness solar energy to facilitate hydrogen evolution from water splitting [4]. Some common photocatalyst materials, such as TiO_2_ [5], ZnO [6,7,8], Ga_2_O_3_ [9,10], CdO [11], CdS [12], MoS_2_ [13], g-C_3_N_4_ [14], BiVO_4_ [15], Ta_2_O_5_ [16], Fe_2_O_3_ [17], metal–organic frameworks (MOF) [18], and covalent organic frameworks (COF) [19], can absorb photons from sunlight and produce electron–hole pairs that drive redox processes, which ultimately cause water molecules to produce hydrogen and oxygen.

Continuous investigation and creation of novel photocatalysts are essential as they hold tremendous promise in dramatically improving the photocatalytic process’s efficiency, selectivity, and stability. These developments will make it more sustainable, economically feasible, and broadly applicable. Within this realm, there has been significant investigation of both iron- and tantalum-based photocatalysts in this area, motivated by the earth’s abundance, cost-effectiveness, and relatively high stability of iron, as well as tantalum’s corrosion resistance, stability in harsh environments, and biocompatibility. Researchers have embarked on an expedition to exploit the distinct benefits of these two elements, resulting in the development of tantalum-doped Fe_2_O_3_ [20,21] and iron-doped Ta_2_O_5_ [22,23], offering significant promise for photochemical water splitting, a critical technique for clean hydrogen generation; for instance, Chang et al. [21] demonstrated a low level of Ta-doped α-Fe_2_O_3_ nanorods for photoelectrochemical water splitting using in situ synchrotron X-ray absorption spectroscopy (XAS). According to the findings, doping α-Fe_2_O_3_ nanorods with low concentrations of Ta enhances its electronic conductivity and charge transfer, overcomes its intrinsic limits, and thus boots its photoelectrochemical water-splitting properties [21]. Iron incorporation into Ta_2_O_5_, on the other hand, can broaden the light absorption range into the visible spectrum, improving solar energy utilization efficiency [22,23]. By using the sol–gel process, Jing et al. [23] prepared Fe-doped mesoporous Ta_2_O_5_, which exhibited enhanced photocatalytic hydrogen evolution ability with a capacity of 20 µmol/h due to a notable shift in light absorption towards the visible light region brought about by the Fe ion doping.

FeTaO_4_ has recently garnered considerable attention in this landscape due to its unique electronic and crystal structure, high chemical stability, and long-term durability characteristics. Moreover, the design of this material intends to combine the capabilities of iron and tantalum, thereby encouraging the development of efficient, sustainable, and economically practical hydrogen evolution technologies and contributing to a greener and more renewable energy landscape. Fanjun’s group reported the development of a nano-sized FeTaO_4_/graphite hybrid composite for usage as an anode material in lithium-ion batteries [24]. The FeTaO_4_ nanoparticles are embedded in graphite nanosheets, resulting in excellent electrochemical performance with higher cycling ability and better capacity than bulk FeTaO_4_ [24]; additionally, Zhang et al. [25] successfully developed Fe_2_O_3_ and FeTaO_4_ core–shell nanorods on an F-doped SnO_2_ (FTO) substrate with notably enhanced photoelectrochemical (PEC) performance. The fabricated nanorods achieved a remarkable photocurrent density of 2.86 mA/cm^2^ at 1.23 V_RHE_ under AM 1.5 G simulated sunlight with an intensity of 100 mW/cm^2^ [25]. 

However, FeTaO_4_ is a relatively unexplored material in photocatalytic hydrogen evolution. Herein, in this work, to address the existing gap in the utilization of FeTaO_4_ in photocatalytic hydrogen evolution, tantalum-oxide-doped FeTaO_4_ composites with circular sheet-like nanostructures were synthesized using a solvothermal-augmented calcination method. The crystalline phase and composition of prepared samples were investigated via XRD, Rietveld refinements, and X-ray photoelectron spectroscopy (XPS) analysis. The optical properties were investigated using UV-visible diffuse reflectance spectra (DRS) and a Tauc plot analysis. Additionally, scanning electron microscope (SEM), transmission electron microscopy (TEM), and ultraviolet photoelectron spectroscopy (UPS) were applied to demonstrate the morphology, crystallinity, and band structure of prepared tantalum–iron composites. Furthermore, the effect of calcination temperatures on the phase composition and particle size of synthesized composites has also been discussed. Additionally, a thorough analysis of their impact on the optical and photocatalytic hydrogen evolution capabilities has been supplied and comprehensively covered, offering insightful information that will help progress this crucial field of sustainable hydrogen production.

## 2. Experimental Section

### 2.1. Materials and Chemicals

Tantalum (Ⅴ) chloride (TaCl_5_, metal basis, 99.8%; Alfa Aesar, Heysham, UK), iron (Ⅲ) chloride anhydrous (FeCl_3_, laboratory reagent grade, ≥97%; Fisher Scientific, Loughborough, UK), N, N-Dimethylformamide (DMF, reagent grade, ≥99%; Honeywell, Seelze, Germany), ammonia solution (NH_4_OH, extra pure, 35%; Fisher Scientific, Loughborough, UK), and terephthalic acid (H_2_BDC, >99%; ACROS organics, Geel, Belgium) were used without further purification.

### 2.2. The Preparation Method of the Ta_2_O_5_/FeTaO_4_

A total of 1 mmol FeCl_3_ and 2.5 mmol H_2_BDC were dissolved into 75 mL of DMF. This mixture was then magnetically stirred for 30 min until a clear brown colour solution was obtained, then 1 mmol TaCl_5_ was added into the solution, changing the colour to yellow. An amount of 10 mL of ammonia solution was then used to adjust the pH of the solution to form a brown suspension. After further magnetically stirring for 30 min, the solution was transferred into a 100 mL Teflon-lined stainless-steel autoclave, sealed, and maintained at 150 °C for 12 h. When the autoclave cooled to room temperature, the precipitate was centrifuged and washed with ethanol several times before being dried in an oven at 70 °C for 12 h. Finally, the resultant powder was individually annealed at 1000 °C for 4 h to generate sample 1000-4 and 1100 °C for 1 h to produce sample 1100-1.

### 2.3. Photocatalytic Hydrogen Evolution Measurement

The photocatalytic hydrogen evolution experiment was conducted in a 530 mL side-irradiation glass reactor with two apertures sealed by two rubber septa under ambient temperature and atmospheric pressure. A Xenon lamp (Newport 66902, 300 W, Newport Spectra-Physics Ltd., Cheshire, UK) was used as the solar light source, which was placed 10 cm distant from the reactor. In total, 0.2 g of prepared tantalum–iron composites were disseminated in 470 mL of aqueous solution containing 35% methanol (*v*/*v*) as the sacrificial electron donor for the measurement. Before experimenting, dissolved oxygen was removed from the solution by constantly injecting nitrogen into the reactor while stirring for 30 min at 80 °C. After the reactor openings were all sealed, a 6 h photocatalytic hydrogen evolution procedure was carried out. For the gas analysis, 0.5 mL of headspace gas was manually injected into the gas chromatograph (GC) once every hour using a micro-injector to measure the hydrogen produced in the reactor.

### 2.4. Stability Study of Produced Samples for Photocatalytic Hydrogen Evolution

Following the measurement of photocatalytic hydrogen evolution, centrifugation was methodically used to separate the powder from the solution. After thoroughly washing with distilled water, the samples were reused without additional treatment. After four repetitions, the final powder was carefully collected and conservatively kept in preparation for the eventual XRD evaluation. 

### 2.5. Photoelectrochemical Performance

The photoelectrochemical performance evaluation was performed using the Metrohm Autolab (PGSTAT302N) electrochemical workstation. A Pt wire was applied for the counter electrode, whereas the reference electrode was made of Ag/AgCl (4 M KCl). A 0.01 M NaOH solution with a pH of 12.8 was used as the electrolyte.

For the fabrication of the working electrode, the screen-printing technique was used. Initially, a solution of 1 g of ethyl-cellulose and 10 mL of ethanal was made, followed by ultrasonic dispersion at 60 °C for 12 h. Following that, magnetic stirring for 30 min produced a clear solution. Later, a slurry was created by blending 0.1 g of the prepared sample with 0.5 mL α-Terpineol and 0.5 mL of the previously described clear solution. This slurry was printed onto a 1 cm × 1 cm fluorine-doped tin oxide (FTO) glass substrate and annealed for 30 min at 450 °C. During the investigation, the transient photocurrent plots were generated over a 300 s duration using the Chrono amperometry (Δt > 1 ms) mode with a 30 s periodic light ON/OFF cycle. Furthermore, electrochemical impedance spectroscopy (ESI) results were collected from 100 kHz to 0.1 Hz, with an amplitude of 10 mV and a bias of 0.23 V vs. Ag/AgCl. 

### 2.6. Characterizations

The crystal structures and phases of the as-prepared powder samples were characterized using a monochromatic Cu-Kα (λ = 0.154 nm) X-ray diffractometer in conjunction with the Bruker D8 Advance XRD. The high-resolution surface morphology and energy-dispersive X-ray mapping (EDX) were investigated separately using a focused ion beam scanning electron microscope (FIB/SEM, FEI Nova 600 Nanolab, Thermo Fisher Scientific, Loughborough, UK) and an FEI Quanta FEG 650 SEM (Thermo Fisher Scientific, Loughborough, UK). Transmission electron microscopy (TEM) and high-resolution transmission electron microscopy (HRTEM) images were captured using a JEOL 2100 instrument (JEOL Ltd., Tokyo, Japan) set to 200 kV accelerating voltage. The XPS and UPS investigations were carried out using a Thermo Fisher Scientific NEXSA (Thermo Fisher Scientific, Loughborough, UK) spectrometer equipped with a micro-focused monochromatic Al X-ray source (72 W) and an X-ray spot size of 400 microns. CasaXPS version 2.3.24PR1.0 software was applied for data analysis, and calibration was performed using the C 1s peak at 284.8 eV. A UV-VIS-NIR Cary 5000 spectrophotometer was used for the UV-visible DRS. The hydrogen generation activity of the produced photocatalysts was investigated using a PerkinElmer GC-580 Clarus gas chromatography (PerkinElmer, Waltham, MA, USA) with an argon carrier gas and a molsieve 5A column coupled with a thermal conductivity detector (TCD).

## 3. Results and Discussion

**Figure 1:**
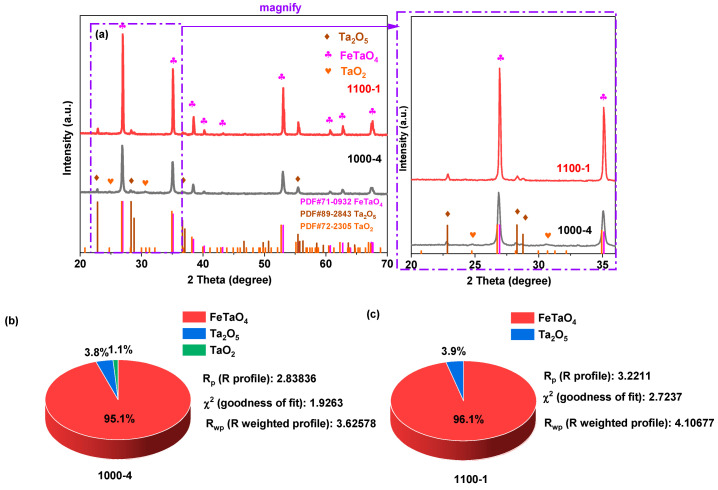
(**a**) XRD patterns; Phase composition and corresponding refined parameters revealed from the Rietveld refinement analysis: (**b**) sample 1000-4 and (**c**) sample 1100-1.

The XRD patterns of prepared samples 1000-4 and 1100-1 are shown in Figure 1a. The diffraction patterns of sample 1000-4 could be attributed to tetragonal phase FeTaO_4_ (space group: P4_2_/mnm, lattice parameters: a = 4.679, b = 4.679, and c = 3.047), orthorhombic phase Ta_2_O_5_ (space group: C2mm, lattice parameters: a = 6.2, b = 3.66, and c = 3.89), and tetragonal phase TaO_2_ (space group: I4_1_/a, lattice parameters: a = b = 13.32 and c = 6.12). When the temperature was raised to 1100 °C for 1 h, no TaO_2_ diffraction peak could be identified, and the peak width became narrower compared to sample 1000-4, which could be attributed to its increased crystallite size and lattice strain. Additionally, a Rietveld refinement analysis was used to determine the phase composition ratio of the prepared samples. As depicted in Figure 1b,c, sample 1000-4 predominantly consists of 95.1% FeTaO_4_, complemented by smaller proportions of 3.8% Ta_2_O_5_ and 1.1% TaO_2_. When the calcination temperature increased from 1000 to 1100 °C, the oxidation of TaO_2_ caused the phase composition to transition from triphasic to biphasic with a composition of 96.1% FeTaO_4_ and 3.9% Ta_2_O_5_. A marginal shift in the major phase composition of the samples, with a difference of only 1%, could be attributed to errors inherent in the Rietveld refinement analysis.

**Figure 2:**
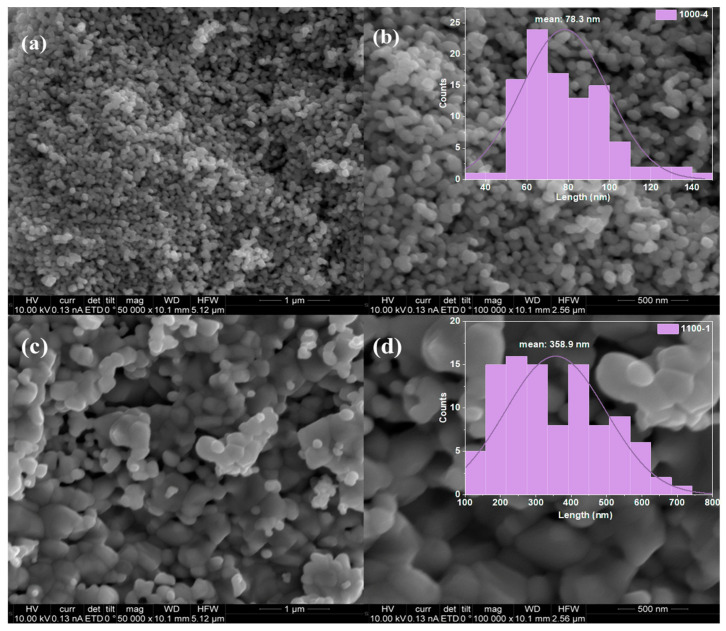
SEM images of the prepared samples and their respective particle size distribution histograms: (**a**,**b**) for sample 1000-4, (**c**,**d**) for sample 1100-1.

SEM investigated the surface morphologies of the produced samples. As shown in Figure 2a,b, the TaO_2_/Ta_2_O_5_/FeTaO_4_ nanoparticles exhibit highly uniform circular sheet-like morphology and are placed into stacks. The particle size distribution histogram in Figure 2b shows that the mean nanoparticle size of sample 1000-4 is around 78.3 nm, with a distribution range of 30–150 nm. The morphology of the prepared Ta_2_O_5_/FeTaO_4_ nanoparticles, shown in Figure 2c,d, becomes a non-uniform nanosheet structure after annealing the precursor at 1100 °C for 1 h, and the nanoparticles’ mean size increases to 358.9 nm, which is four times larger than that of sample 1000-4. Moreover, thermal treatment introduced the Ostwald ripening phenomenon [26,27], which causes particle size to increase because of surface diffusion and adhesion to pre-existing particles. 

**Figure 3:**
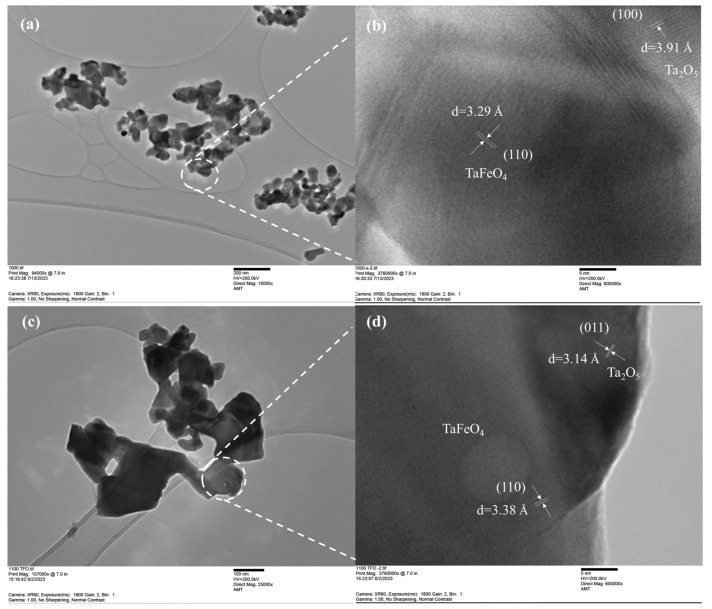
TEM (**a**) and HRTEM (**b**) images of sample 1000-4; TEM (**c**) and HRTEM (**d**) images of sample 1100-1.

TEM and high-resolution TEM were applied to investigate the produced samples’ morphological and internal structural analyses. The TEM images, displayed in Figure 3a,c, revealed that sample 1000-4 had a sheet-like morphology with particle size less than 150 nm, while sample 1100-1 displayed significantly larger dimensions than sample 1000-4, confirming and solidifying the SEM findings. The HRTEM image in Figure 3b also shows distinct lattice fringes with interplanar spacings of 3.29 Å and 3.91 Å, corresponding to the (110) and (100) planes of FeTaO_4_ and Ta_2_O_5_, respectively. The clear lattice fringes in Figure 3d have interplanar spacings of 3.38 Å and 3.14 Å, respectively, consistent with the (110) and (011) planes of FeTaO_4_ and Ta_2_O_5_. Moreover, irregular regions on the surface of the produced FeTaO_4_ and Ta_2_O_5_ crystals in the form of discontinued lattice fringes suggested the presence of defects, which could be attributed to oxygen vacancies within the crystal lattice.

**Figure 4:**
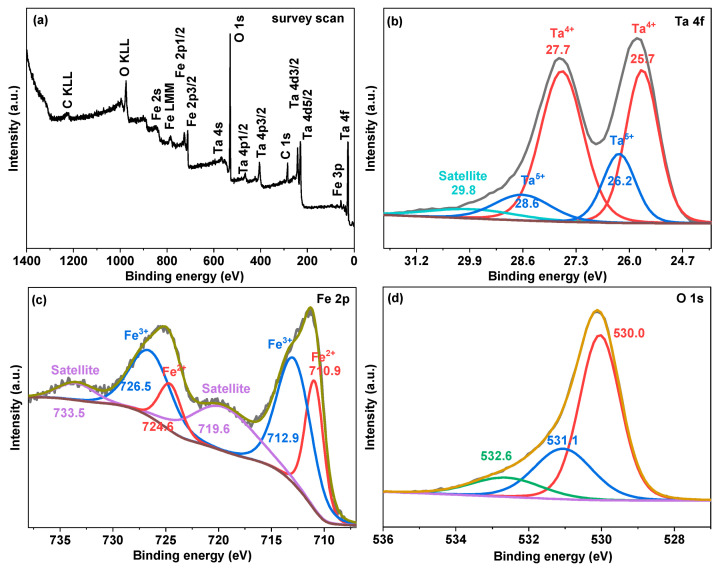
(**a**) XPS survey scan spectrum of sample 1000-4, and the corresponding high-resolution spectra for (**b**) Ta 4f, (**c**) Fe 2p, and (**d**) O 1s.

The XPS investigated the produced samples’ surface composition and chemical states. All the results were calibrated using the C 1s electron at 284.8 eV as the reference point. The survey spectrum depicted in Figure 4a reveals that the prepared sample of 1000-4 is composed of Ta, Fe, and O. The two spin-orbit peaks in the high-resolution spectrum of Ta 4f in Figure 4b correspond to Ta 4f_7/2_ and Ta 4f_5/2_, respectively. Each of the Ta 4f_7/2_ and Ta 4f_5/2_ core levels could be deconvoluted into two peaks. The 26.2 and 28.6 eV peaks could be ascribed to Ta^5+^ in FeTaO_4_ and Ta_2_O_5_. 

In contrast, the two peaks at lower binding energies, 25.7 and 27.7 eV, could be attributed to Ta^4+^ in TaO_2_ and other low valance state Ta^4+^ species introduced by oxygen vacancies [28]. The high-resolution Fe 2p spectrum in Figure 4c shows four peaks: Fe 2p_3/2_ at 711.3 eV, Fe 2p_1/2_ at 725.2 eV, and the other two satellite peaks at 719.6 and 733.5 eV. After fitting, both the Fe 2p_1/2_ and Fe 2p_3/2_ peaks could be deconvoluted into two peaks: the peaks at 712.9 and 726.5 eV can be assigned to Fe^3+^ species in FeTaO_4_, while the other two peaks at 710.9 and 724.6 eV could be linked to Fe^2+^ species [29,30]. Additionally, the O 1s high-resolution spectrum in Figure 4d can be resolved into three peaks: lattice oxygen species (O^2−^) at 530.0 eV, oxygen vacancies in the composite at 531.1 eV, and absorbed oxygen species such as O_2_, H_2_O, and CO_2_ at 532.6 eV [31]. 

**Figure 5:**
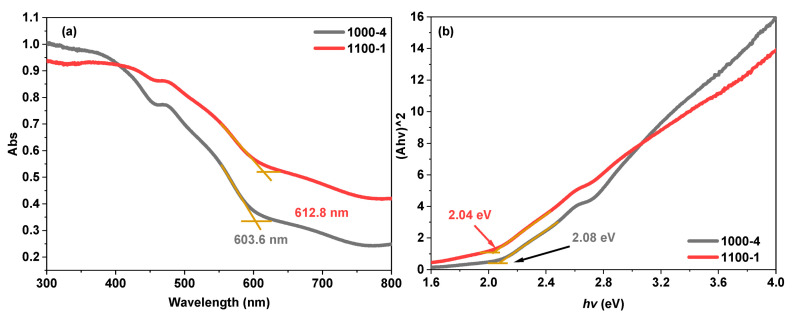
(**a**) UV-vis DRS spectra and (**b**) Tauc plot analyses for bandgap determination of sample 1000-4 and 1100-1.

The UV-vis DRS spectroscopy investigated the light absorption behaviour of prepared samples, and the findings are shown in Figure 5a. The absorption edge of sample 1000-4, with a substantial rise in absorbance, is located at 603.6 nm, while sample 1100-1 shows a significant increase at 612.8 nm. A red-shift of the absorption band edge of produced materials with increasing temperature from 1000 to 1100 °C could be attributed to the influence of quantum confinement effects becoming less relevant as the particle size increases [32]. According to the examination of the Tauc plots in Figure 5b [33], the corresponding bandgap energies for samples 1000-4 and 1100-1 are calculated to be 2.04 eV and 2.08 eV, respectively.

**Figure 6:**
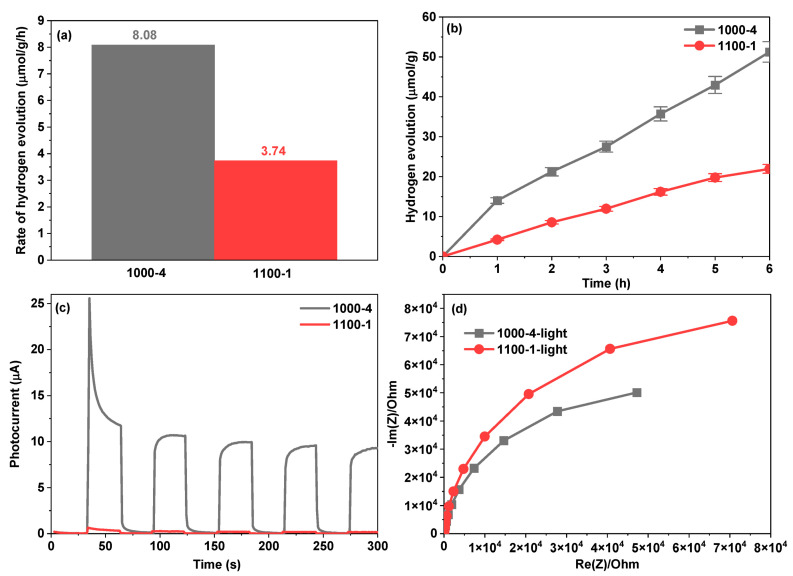
(**a**) Photocatalytic hydrogen evolution rates, (**b**) time-dependent photocatalytic hydrogen evolution performance, (**c**) transient photocurrent plots, and (**d**) ESI Nyquist plots of produced samples 1000-4 and 1100-1.

Under solar light irradiation, the prepared 1000-4 and 1100-1 samples’ photocatalytic hydrogen evolution activities were evaluated in a methanol–water solution. As illustrated in Figure 6a, sample 1000-4 demonstrated favourable hydrogen evolution ability with a rate of 8.08 µmol/g/h, surpassing the performance of other photocatalysts listed in Table 1. This rate is 2.16 times higher than 1100-1, which yielded 3.74 µmol/g/h. Additionally, after six hours of solar light irradiation, sample 1000-4 achieved a hydrogen evolution amount of 51.24 µmol/g, surpassing sample 1100-1, which produced 21.94 µmol/g. The enhanced performance in photocatalytic hydrogen evolution could be attributed to the more significant number of active sites resulting from a higher surface-area-to-volume ratio compared to the larger nanoparticles generated in sample 1100-1. Moreover, the transient photocurrent curves and the electrochemical impedance spectroscopy (ESI) plots depicted in Figure 6c,d indicate that sample 1000-4 exhibits superior photocurrent response and lower charge transfer resistance compared to sample 1100-1. These findings demonstrate that sample 1000-4 efficiently enhances charge carrier separation and transfer processes.

**Figure 7:**
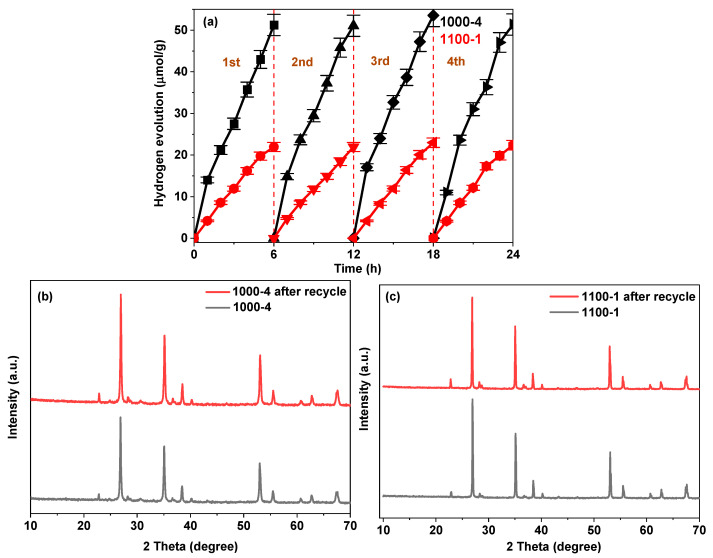
(**a**) Photocatalytic stability plots for hydrogen evolution rate; XRD patterns of sample 1000-4 (**b**) and 1100-1 (**c**) before and after the reaction.

A photocatalytic test for hydrogen evolution production was conducted over four consecutive cycles without any catalyst regeneration steps to evaluate the photocatalytic stability of the produced samples. Specifically, the suspension was extracted from the photoreactor and centrifuged to recover the photocatalyst. After thorough washing with distilled water, the samples were reused without additional treatment. As shown in Figure 7a, both samples demonstrate outstanding durability in the photocatalytic hydrogen evolution reaction. The results presented in Figure 7a indicate that even after undergoing four consecutive cycles of photocatalytic hydrogen evolution reactions, there is no discernible decrease in the overall amount of hydrogen evolution. This substantiates the exceptional stability exhibited by samples 1000-4 and 1100-1. Furthermore, comparing XRD patterns before and after the photocatalytic reaction (Figure 7b,c) reveals that the crystal structure remains unaltered. This further validates the stability of the prepared samples. 

**Figure 8:**
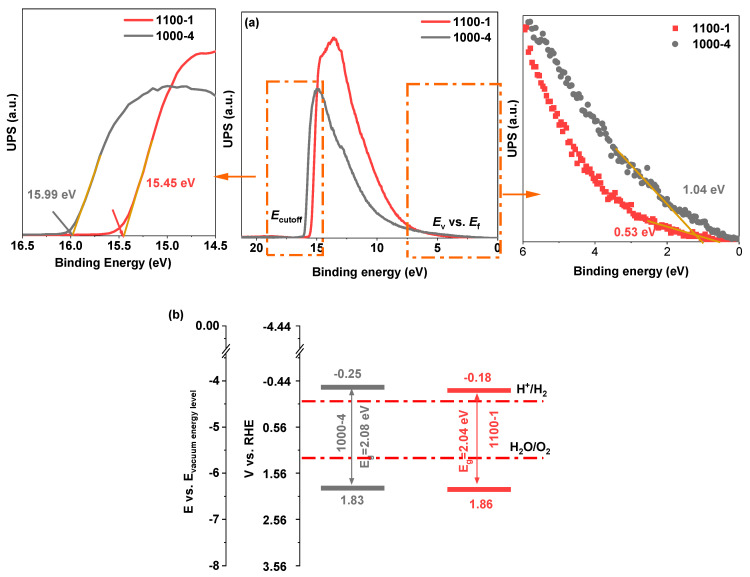
The produced samples’ (**a**) UPS spectra and (**b**) band structure diagram.

The valence band edges for the 1000-4 and 1100-1 samples were calculated using UPS, as shown in Figure 8a, and were found to be 6.27 eV and 6.3 eV vs. vacuum level, respectively. These values were derived by deducting the width of the UPS spectra from the excitation energy (21.22 eV). Additionally, as illustrated in Figure 8b, the bandgaps and band positions measured provided insight into the band structures of the produced samples. The conduction band (CB) potential of sample 1000-4 is −0.25 V vs. RHE, exhibiting a more negative value than the −0.18 V of sample 1100-1 and displaying its superior ability of photocatalytic hydrogen evolution. The more negative CB potential of sample 1000-4 increases the driving force for electron transfer. It provides the necessary energy for efficient electron transfer during water reduction, making it easier to complete photocatalytic hydrogen evolution. This more suitable band structure could be attributed to the quantum confinement effect, which generated an energy level shift by controlling the size of the nanoparticles to below 100 nm. 

**Figure 9:**
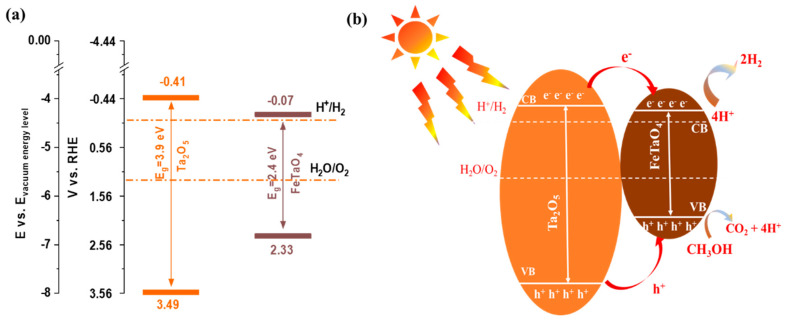
(**a**) Energy band positions of Ta_2_O_5_ and FeTaO_4_ and (**b**) corresponding schematic diagram depicting the photocatalytic hydrogen generation process.

Band potential determination is a vital parameter in the precise design of the heterojunction structure. It is critical to understand the complicated mechanism of regulation of photogenerated electron and hole transfer and separation, which are crucial to photocatalytic hydrogen evolution. Given that our method did not yield pure phases of FeTaO_4_ and Ta_2_O_5_ directly, we have meticulously gathered the required values in Table 2 from the previous scientific literature. For visual reference, the corresponding band structure is shown in Figure 9a. Based on the results mentioned above, a possible mechanism was presented in Figure 9b to explain better the charge transfer process in these tantalum–iron composites. As illustrated in Figure 9b, Ta_2_O_5_ and FeTaO_4_ absorb solar radiation to produce electron–hole pairs [41,42]. The photo-generated electrons then transfer from the valence band (VB) to the conduction band (CB) of Ta_2_O_5_ and FeTaO_4_, while the holes remain in the VB [41,42,43]. Upon the formation of the heterojunction at the interface of Ta_2_O_5_ and FeTaO_4_, the photo-generated highly energetic electrons within Ta_2_O_5_ undergo a transfer to the CB of FeTaO_4_; at the same time, the excited holes will transfer to the VB of FeTaO_4_ due to their distinctive band position [42,43,44,45]. This charge carrier migration is principally contributed by the more positive CB and the more negative VB of FeTaO_4_. The electrons in the CB of FeTaO_4_ are likely to anticipate reacting with photons from water to form hydrogen. At the same time, the holes in the VB of FeTaO_4_ can oxidize methanol, which serves as a sacrificial electron donor, to produce photons and CO_2_ [44]. The metallic TaO_2_ in sample 1000-4 acts as a co-catalyst by providing additional electrons and a surface for catalysing photon reduction into hydrogen, further enhancing the photocatalytic hydrogen evolution process [46].

## 4. Conclusions

In this work, we successfully prepared circular sheet-like structural tantalum–iron composites and investigated their potential prospective use as efficient photocatalysts for hydrogen evolution. The systematic research of different calcination temperatures (1000 °C and 1100 °C) provided valuable insights into how these conditions influence the composites’ phase composition and particle size, allowing for improved control over their photocatalytic properties. Notably, sample 1000-4, composed of a TaO_2_/Ta_2_O_5_/FeTaO_4_ heterojunction composite, exhibited an impressive photocatalytic hydrogen evolution ability, surpassing the performance of sample 1100-1 (Ta_2_O_5_/FeTaO_4_) by more than twofold. The increased photocatalytic effectiveness found in samples 1000-4 can be attributed to several essential factors. Upon comprehensive analysis of the optical properties and band structure, it is posited that the formation of heterojunctions could amplify the photoelectrochemical properties and photocatalytic performance. This enhancement seems to be attributable to an augmentation in the number of active sites, an optimized band structure, and a heightened charge separation efficiency. Our results underscore the significance of judicious material design and highlight the potential of tantalum–iron heterojunction composites as catalysts. Such materials hold promise in advancing the renewable energy landscape, particularly within the solar hydrogen research domain.

## Figures and Tables

**Table 1:** Metal oxide photocatalysts for photocatalytic hydrogen evolution.

Photocatalysts	Hydrogen Evolution Rate (µmol/h)	Light Resource	Ref.
P25	6.4	Ultraviolet light	[34]
Carbon-incorporated Co_3_O_4_	0.85	Solar light	[35]
InVO_4_	5	Ultraviolet light	[36]
InTaO_4_	4	Ultraviolet light	[37]
BiTaO_4_	4	Ultraviolet light	[38]
NaTaO_3_: La/Cr	4.4	Ultraviolet light	[39]
Cu_2_O/WO_3_	1.9	Ultraviolet light	[40]

**Table 2:** Band gap energy (*E*_g_), valance band (*E*_v_) band position, and conduction band (*E*_c_) position.

	*E*_g_ (eV)	*E*_c_ (eV)	*E*_v_ (eV)	Ref.
Ta_2_O_5_	3.9	−4.03	−7.93	[47]
FeTaO_4_	2.4	−0.16	2.24	[25]

## Data Availability

Data are available upon request to the corresponding author.
